# Deep Q-Learning in Robotics: Improvement of Accuracy and Repeatability

**DOI:** 10.3390/s22103911

**Published:** 2022-05-21

**Authors:** Marius Sumanas, Algirdas Petronis, Vytautas Bucinskas, Andrius Dzedzickis, Darius Virzonis, Inga Morkvenaite-Vilkonciene

**Affiliations:** Department of Mechatronics, Robotics and Digital Manufacturing, Vilnius Gediminas Technical University, 10223 Vilnius, Lithuania; marius.sumanas@vilniustech.lt (M.S.); algirdas.petronis@vilniustech.lt (A.P.); vytautas.bucinskas@vilniustech.lt (V.B.); andrius.dzedzickis@vilniustech.lt (A.D.); darius.virzonis@vilniustech.lt (D.V.)

**Keywords:** machine learning, positioning errors, robotics, deep q-learning, reinforced learning, robot operating system ROS

## Abstract

Recent industrial robotics covers a broad part of the manufacturing spectrum and other human everyday life applications; the performance of these devices has become increasingly important. Positioning accuracy and repeatability, as well as operating speed, are essential in any industrial robotics application. Robot positioning errors are complex due to the extensive combination of their sources and cannot be compensated for using conventional methods. Some robot positioning errors can be compensated for only using machine learning (ML) procedures. Reinforced machine learning increases the robot’s positioning accuracy and expands its implementation capabilities. The provided methodology presents an easy and focused approach for industrial in situ robot position adjustment in real-time during production setup or readjustment cases. The scientific value of this approach is a methodology using an ML procedure without huge external datasets for the procedure and extensive computing facilities. This paper presents a deep q-learning algorithm applied to improve the positioning accuracy of an articulated KUKA youBot robot during operation. A significant improvement of the positioning accuracy was achieved approximately after 260 iterations in the online mode and initial simulation of the ML procedure.

## 1. Introduction

In complex manufacturing, articulated robots are chosen more frequently because of their advantages: multidegree-of-freedom (multi-DOF), wide working space, offline programming (OLP) capability, and high dexterity [1]. OLP significantly saves operating time and investment in the development of complex-shaped objects for the production process [2]. Nevertheless, OLP often does not ensure the required accuracy and repeatability; even using the most advanced robotic system, the motion parameters, namely, coordinate, velocity, acceleration, deceleration, and required positioning tolerance, have to be adjusted manually before operation [3]. However, sometimes even the final adjustment does not provide the desired accuracy due to the actual positioning errors. The value of errors depends on the robot characteristics and manipulation task [4]. Errors that depend on robot characteristics include inaccuracies in the assembly, transmission gear backlashes, and arm compliance [5]. In addition, kinematic mistakes occur due to serially connected links that accumulate errors related to drives, mechanisms, and joints from the robot base to the end effector [6,7,8,9]. Manipulation task performance depends on environmental conditions, applied load, stiffness of mounting surface, and tool characteristics, such as rotation speed, angle, and even lubrication [10]. Moreover, robot movement trajectory and setpoint position in the workspace influence its positioning accuracy [11]. Additionally, the robot’s movement is always affected by dynamic processes. Therefore, such a complex origin of positioning errors limits the implementation of common error compensation techniques [12,13,14,15,16,17,18]. Therefore, new and reliable methods, such as visual recognition systems and machine learning (ML) algorithms, can be applied to improve robot positioning accuracy and repeatability during operation [19,20,21,22].

One of the widely used ML algorithms to control physical equipment is the deep q-learning algorithm, which belongs to the reinforcement learning ML type [23]. It allows a robot to find the best positioning accuracy through trial-and-error interactions with the environment rather than requiring positive or negative labels [24,25,26,27,28,29]. As a part of the general robotic operation optimization technique, it gradually finds the best positioning method and seeks to discover the most considerable cumulative reward value in each iteration There are two main advantages of using the deep q-learning algorithm in an industrial robot case: the possibility to introduce gathered live video data into it [30,31] and the possibility to avoid the commonly known ML problems such as overfitting. The principle of deep q-learning is based on the perception of the environment [32], the state of the robot, and respective actions to achieve the maximum reward. Previous research proved this algorithm’s suitability for defining the robot tool position using visual data, finding the best combination of robot motion parameters, and generating output results with corrected setpoint coordinates [26,29,33].

Since online robot training is a time-consuming procedure, the values of positioning accuracy can be determined using ML simulation for actual cases. The simulation lasting less than five minutes replaces physical experiments lasting more than five hours. The convenience of the simulation is that it only requires a computer (except when it is necessary to collect input data from the real robotic system). However, any simulation cannot perfectly represent real-world conditions due to assumptions and simplifications used to describe various unknown or undefined physical effects. Therefore, even if the algorithm works well, simulation results require validation from experiments performed in operational conditions. Consequently, simulation is an excellent method to select the most promising combinations of algorithm parameters for testing in the real world.

The main aim of this paper is to present a methodology for the improvement of industrial robot accuracy and repeatability using the deep q-learning algorithm with optimal parameters. The methodology was evaluated by comparing simulations and experiments using the KUKA youBot articulated robot as a workbench.

## 2. State-of-the-Art

This section reviews the machine learning-based methods used to improve the performance of mechatronic and robotic systems or their components. From the perspective of industrial robotics, the main ML implementation fields and goals are: sensor data analysis—to increase object detection/recognition accuracy; tool path generation—to optimize motion trajectory or energy consumption; and robot calibration and positioning errors analysis—to save operational time and increase positioning accuracy.

### 2.1. Trajectory Generation and Its Accuracy

Trajectory generation is an essential task for robots and robotic systems operating in all possible applications. Trajectory defines robot movement quality in terms of the accuracy of static points at the end of the trajectory, intermediate points as waypoints within, and acceleration levels during the entire movement cycle. Good trajectory uses minimum possible intermediate points, defines paths using higher-order curves, and uses internal robot interpolation facilities as the fastest and most efficient resource. Issues of accuracy cover the entity of volumetric (or, in case, planar) coordinates of the target as a basis for error definition at the actual robot position. Angular coordinate deviation questions are often left behind except when manipulation tasks raise the requirement to ensure precise tool orientation [34,35].

The accuracy of a robot’s trajectory and the required computational resources strictly depend on the trajectory generation method. Recently, more and more attention is being paid to the automatic trajectory generation by copying human movements, for example, in surgery, where movement trajectory is of vital importance. Wang and Majewicz Fey developed a special ML procedure that implements a neural network for robot trajectory generation [36]. The proposed method uses human gesture analysis as input and successfully transforms it into a final robot motion. During experimental validation, highly competitive accuracies were achieved of 92.5% for suturing, 95.4% for needle passing, and 91.3% in the knot-tying operations.

A summarized overview of various ML approaches for the improvement of robot positioning accuracy and generation of trajectory is provided in Table 1.

An overview of the references shows that there are three main directions of ML implementation in the field of positioning accuracy enhancement and trajectory generation:

- The development of new methods for manipulator calibration to compensate for the positioning errors regardless of their nature. It is performed similar to our methodology.
- Implementation of ML into the robot controller to solve kinematic problems occurring in complex structure manipulators where traditional methods are not suitable or require a lot of computational resources.
- Implementation of ML into the trajectory generation process to enhance or generate input data to optimize generated trajectory.

### 2.2. Object Grasping

Using a robot as a manipulator, a device for pick-and-place tasks, and a general device with a changeable load or end-of-arm tool embraces a general problem of grasping. The variety of objects from solid to almost liquid state and their different geometric shapes raises the requirements for object manipulation. However, this issue faces limitations such as maximal acceptable forces or accelerations during the motion. Solutions existing in the grasping area developed a tree-like structure of standard objects and assigned solutions such as two- or three-finger grabbers. Nevertheless, individual problems raise tasks for original designs and conceptions. The implementation of ML procedures recently covers grabbing parameters, but, unfortunately, ideas and applications must be generated only by human beings. ML in this area can adjust grabbing parameters to the optimal values. 

The manipulation of complex geometry objects that slightly change their position or orientation due to grasping action is a difficult task [22]. Typically, directions and amplitudes of such movements are chaotic and hardly predictable due to arising uncertainties. The authors raise the hypothesis that concerning the required positioning accuracy, the machine learning-based classification method and computer vision system could be used to define the object’s actual position after grasping. Such a solution could provide crucial information for real-time movement trajectory correction. A similar object recognition problem is mentioned in [45], where a composite structure inspection system consisting of an industrial robot and laser profilometer is described. The robot actuates the profilometer and quickly scans the part’s profile, providing profile height data as a grayscale image. A convolutional neural network detects defects and assigns them to a certain class. Product assignment to a particular class defines the required adjustments in the binding material allocation algorithm, which typically results in trajectory changes of the robot or other device placing the binder.

Luo et al. [46] presented a reinforcement learning-based solution for force/torque control to assemble a tight-fit gear wheel set. Experiments showed that using reinforcement learning, the robot provided fewer assembly errors due to part misalignment. Lie et al. [47] implemented the least squares support vector machines method for image classification to sort coal and gangue. The proposed system reached the identification accuracy of coal 88.3%, and the identification accuracy of the gangue sample was 90.0% with 0.130 s average total time for one sample recognition. Reviews by Li et al. [48] and Bai et al. [49] analyze the research progress in implementing machine learning for robotic grasping, including aspects of machine vision or tactile feedback. A condensed review of the related research is provided in Table 2.

The result of the performed review reveals three main directions on how ML can be used in the grasping process:

- The combination of ML and computer vision ensures more precise object detection and recognition as well as a more accurate definition of optimal grabbing position and grabber orientation.
- ML can be used to analyze and process position or force feedback signals when it is necessary to adjust the final robot tool position for successful grasping.
- ML significantly improves the manipulation process of unstable objects that can change their position or shape due to the low stiffness, grasping impact, or gravity forces.

### 2.3. Sensor and Instrumentation

The intelligent application of robots and robotic systems requires sensors and their data processing systems. Such systems recently armed with machine learning algorithms can achieve high efficiency and early detection of issues. The implementation of ML procedures can also sharpen these systems by increasing their accuracy or resolution. The use of the sensors allows the creation of systems with artificial (synthetic) ontology, which opens a space for further application of ML procedures. Instrumentation in robotics covers fault detections of the system, early detection of defects, failure prevention, and other issues required for successful task execution. A big issue in robotics is applications with virtual and augmented reality, allowing one to enhance the approach of synthetic ontology. This field of research lays on the frontier of applied science, and achievements there will bring significant changes in robot perception.

Ribeiro et al. [53] used ML to increase the accuracy of human movement trajectory tracking using inertial measurement units. Their proposed method defines zero velocity and evaluates the regression of translations in periods of movement. Xiao et al. [54] proposed the implementing of ML to decrease IMU tracking errors by learning typical error patterns and later compensating for them. As a result, the obtained trajectory could be transformed easier and faster into robot motion commands.

Cheng et al. in [55] proposed a fault prediction method for industrial robots. It measures the electrical current used by the motors and analyzes it in time and frequency domains. The main diagnostic parameters are the magnitude fluctuation index and signal-to-noise ratio. The magnitude fluctuation index is extracted in the time domain using Hilbert transformation, and the signal-to-noise ratio is defined as the ratio between frequencies in the spectrum obtained by the short Fourier transformation. The Gaussian mixture model detects failure by clustering the obtained parameters as normal and faulty.

The outcome of the review of research focused on sensors and instrumentation in robotics is listed in Table 3.

The analysis of references related to ML-based sensing and instrumentation in robotics emphasizes that the mainstream in this field is human movement trajectory tracking and its transformation into the robot control program. Another actual research direction is the implementation of ML for internal robot parameter monitoring to predict possible faults and unwanted impacts from the environment, for example, contact with other objects. 

The provided analysis of ML applications in the field of robotics covers specific areas mentioned in the highlighted references that focus on various aspects but mainly result in the increase in accuracy, efficiency, or functionality. Nevertheless, this area is not limited by these research items. The wide variety of robotic tasks creates a considerable space for unique and individual solutions.

## 3. Materials and Methods

### 3.1. Deep Q-Learning-Based KUKA YouBot Control Method

**Figure 1:**
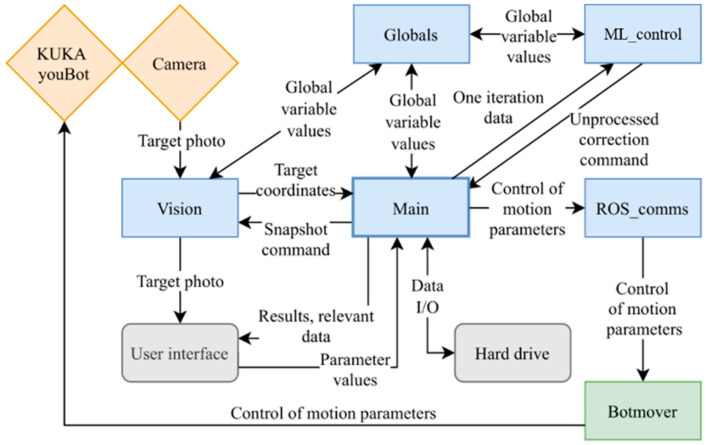
Control system scheme: blue—Python programs, green—C++ program, yellow—physical peripherals, grey—physical computer elements and accessories.

The KUKA youBot articulated robot [59] controlled by a unique software platform consisting of six merged modules (Figure 1) was used in our research. All software modules, mainly written in Python language, operate simultaneously and perform individual functions. Communication with external devices is performed through additional drivers, libraries, and robot operating system (ROS). The latter communicates with the robot using dedicated drivers and libraries provided by the manufacturer.

The created ML_control module contains the deep q-learning algorithm [60]. It receives the values of global variables from the Globals module, which synchronizes Globals’ variable values among several Python programs running on the control system. The ML_control module communicates with the Main, accepting input values and sending the ML algorithm’s output value.

The user interface was realized within the Main module, which controls the flow of the experiment. The Main module also includes experiment and simulation algorithms and the data input/output functions. The Main sends the command to the Vision module, which takes a photo of the target using a digital microscope, identifies the target, calculates its center coordinates, and sends them back to the Main.

The Botmover module gives low-level access to the robot control parameters and allows control of the KUKA youBot by an external program. The primary function of this module is to control robot joint angles based on coordinate requirements received from the Main via ROS_comms. Once a set of coordinates and a command to move to that position are received, angle values for each joint are calculated by solving the inverse kinematic task. Then, by employing youBot libraries and drivers, the joint angle data are sent to the robot to perform the corresponding motion. Such a method enables close to real-time control of the robot from the Main module. The Botmover runs in an infinite loop until interruption by the user or another program.

Communication between the Botmover and other programs runs in asynchronous mode. It is based on the publisher–subscriber principle and is realized via means and libraries of ROS. The ROS_comms module acts as an intermediary between the Botmover program written in C++ and the Python programs. It enables full control of the robot parameters and movements directly from other Python applications.

### 3.2. Implementation of the Method 

**Figure 2:**
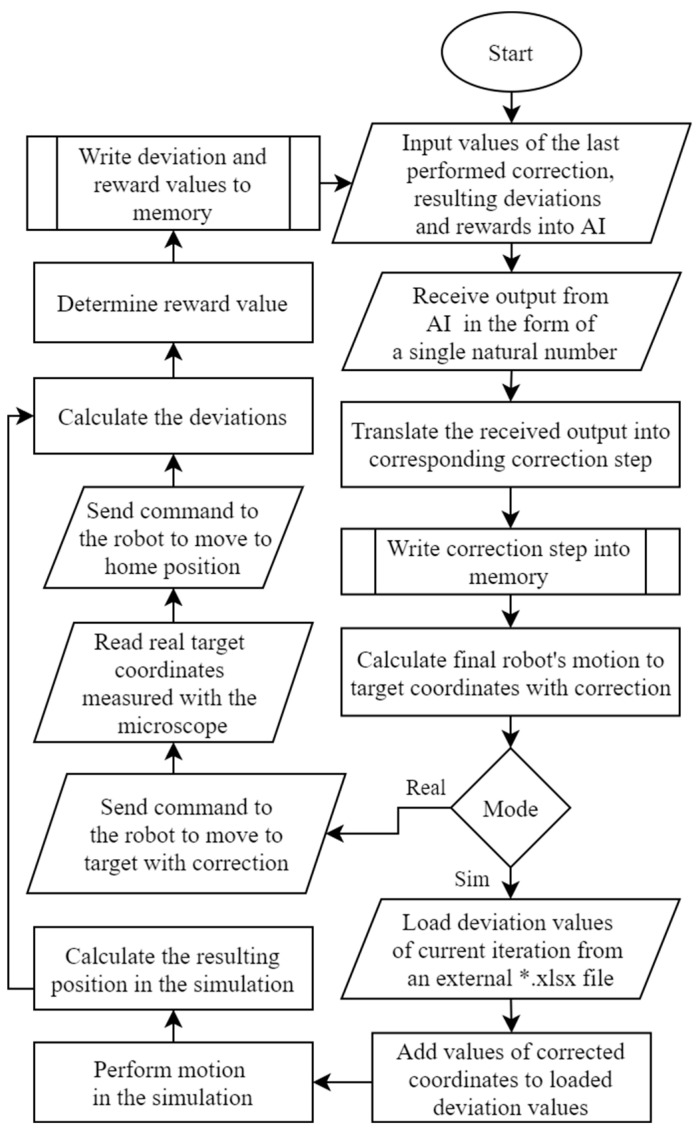
Principle of experimental and simulation algorithms in Main module (from Figure 1).

The simulation and experiment algorithm (Figure 2) running in the Main module is a cycle performing certain functions that repeat many iterations (400–4000). The cycle begins by inputting five values from the previous cycle into the ML. If the cycle runs for the first time, the parameter’s value is zero. In the other cases, the algorithm takes the correction step and the deviation values in the y and z directions from the previous cycle.

The output from the ML goes in the form of a single natural number, thus converting it into the appropriate correction step stored in the memory. The correction step is a correction performed in one iteration and exists in the form of a vector (value and direction). Firstly, this is where an array of vectors is created containing all defined vectors. Then, the accepted ML output is assumed to be an index of that array. Each ML output value is assigned with the corresponding correction step. The vector components of the correction step are defined with the coordinates of the target in space to obtain the corrected coordinates.

The goal of the algorithm is to obtain corrected coordinates. The correction steps are cumulative. They continue to add the results of the current iteration to the results of all previous iterations. After defining the corrected coordinates, the robot receives the command to execute them in real-time. The Vision module takes the target picture, calculates the actual target coordinates, and sends a command to return the robot to the “home” position. The actual coordinates of the target are compared with the theoretical ones to determine the deviation values and directions.

After determining the deviations, the ML algorithm activates the reward function, which determines the value of the reward depending on the obtained deviations value. The value of the reward indicates to the algorithm how much the performed action corresponds to the desired result.

A positive reward indicates that the current iteration achieved the desired result. The magnitude of the reward is proportional to the result achievement level. A positive reward is given as output when one of the two conditions is fulfilled: (I) the deviation found in the current iteration must be smaller than in the previous iteration; (II) the deviation in the current iteration is less than half of the average deviation when the robot moves without correction. A negative reward is received when: (I) the measured deviation in the current iteration is greater than or equal to the deviation in the previous iteration; (II) or the threshold is exceeded (correction value is so high that any deviation compensation is impossible).

Such a reward function motivates the algorithm to perform actions that would reduce the deviations in each iteration until, in an ideal situation, a specific optimal point (average deviation reduced by half) is reached. However, due to the stochastic nature of some sources of positioning errors, it would be very difficult or impossible to reach such a point. Nevertheless, it is useful to have such a point as the goal is an ideal deviation value that must fit as close as possible.

The resulting deviation and reward values are stored in memory at the end of each iteration. Afterward, a learning cycle is restarted for a new iteration.

For speed and convenience, the Main module can also perform simulations with or without the use of collected real data. The simulation uses most of the same blocks of the Main code and the actual experiment code (Figure 2). The difference is that instead of sending commands to the robot and the camera, the simulation uses data collected experimentally when the robot was moving thousands of iterations without correction. These data represent the actual positioning accuracy of the robot. Such a dataset gives a possibility to use it in an unlimited number of simulations. Moreover, it minimizes the required number of iterations by loading the algorithm memory with real data before performing the online training.

### 3.3. Methodology of the Research

By using data representing experimentally obtained actual coordinates when the robot moves without correction, many simulations were performed in advance to speed up the experiments and define the best combination of algorithm parameters.

The deep q-learning algorithm with parameters providing the most seemingly successful results was further tested experimentally.

**Figure 3:**
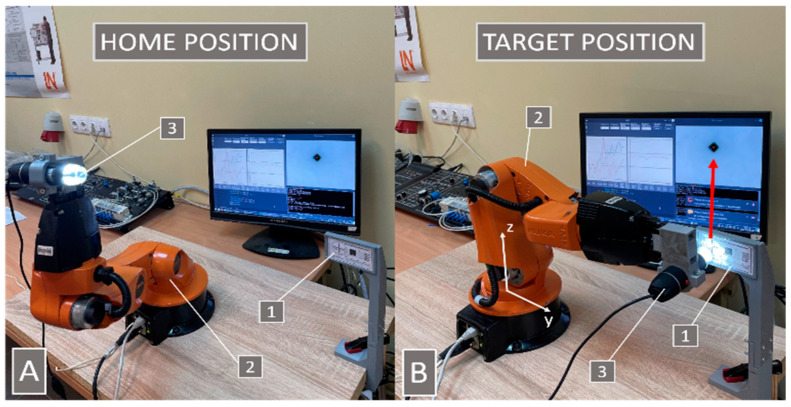
Robot “home” position (**A**) and the target position (**B**). The robot moved between these two positions during each cycle of the experiments. 1—target, 2—robot, and 3—microscope.

All experiments were performed at maximum operating speed when all joints were rotating at 90°/s to test the worst possible conditions at which the accuracy is mainly affected by inertial forces. The trajectory of the robot movement, the “home”, and target positions (Figure 3) were chosen so that all robot joints would move by similar angles during movements.

The experiment began by finding the theoretical target. The robot moved to the “home” and “target” positions 100 times, while the Vision module defined the target center coordinates and sent them to the ML_control memory. The ML_control calculated the target’s average coordinates, further used in the training procedure algorithm.

The algorithm was trained using parameter values obtained from simulations. The robot moved from the “home” position to the target position with the target coordinate correction. A digital microscope took a picture of the target and sent it to the Vision module to detect the target position in the picture and define its center coordinates. The obtained coordinates were transferred to the Main module, which processed the data and wrote them to memory. The Main module calculated the deviation in *y* and *z* directions and wrote data into memory. Then the procedure activated the reward function and saved the determined reward for the deep q-learning algorithm in computer memory. Horizontal (*y*), vertical (*z*), and absolute deviations, as well as previous correction steps, were transferred into the machine learning algorithm. The algorithm calculated the values of the coordinate corrections for the next iteration. In the simulation run of 4000 iterations, experimental tests stopped after completing 800 iterations since further training did not give any improvement.

## 4. Results

### 4.1. Results of the Simulation

The standard deep q-learning algorithm has four main parameters: activation function, optimizer, replay memory, and temperature [49]. The activation function defines the algorithm’s output for a given input or set of inputs. An optimizer is a parameter that controls interconnections among neurons (synapses) and affects how the final algorithm works over a long period. The replay memory is a function that allows the algorithm to use the previous experience to decide on the present. Temperature is a parameter that regulates the exploration–exploitation ratio. The efficiency of the deep q-learning algorithm depends on the combination of these parameters.

The appropriate algorithm configuration and its parameters were obtained from the simulation procedure. The ML algorithm made coordinate corrections to compensate for the positioning errors, thus improving the robot’s positioning accuracy after a number of training cycles of the machine learning algorithm. The number of training cycles for the machine learning algorithm was small enough to make training practical in terms of time (e.g., the total training took no more than a few hours). After developing the optimal algorithm, multiple variations of the parameters were tested and evaluated in the simulations. By varying each parameter, tens of 4000 iteration length simulations were performed to find the best combination of their values.

**Figure 4:**
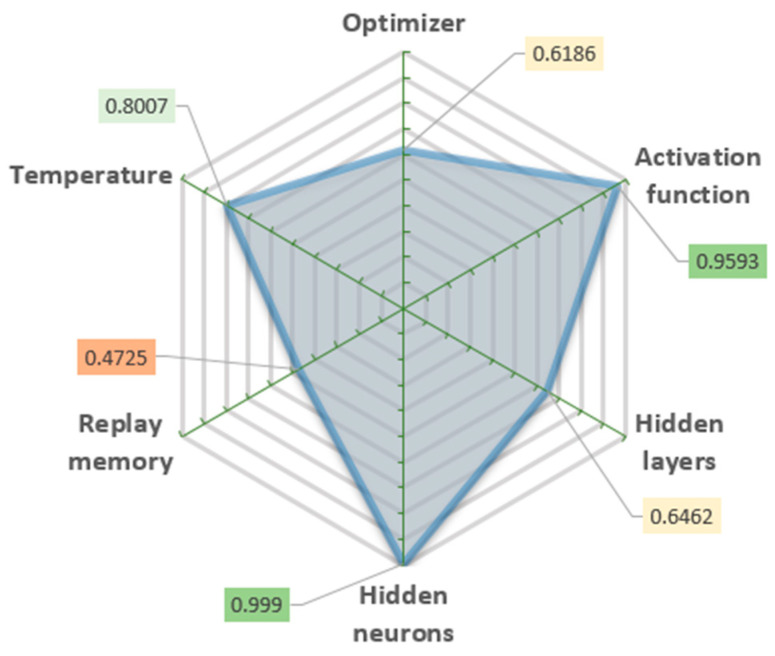
Algorithm configuration parameters and their significance.

The quality of the defined parameter combinations was evaluated by the standard deviation of the mean error values of the last 300 iterations (Figure 4) and the stability of the long-term process. These values show how strongly the results vary when a certain parameter is changed. The lower standard deviation value means that the smaller parameter change affects the result.

The determined optimal combination of parameters shows that an extensive artificial neural network is not required for this purpose, but an extensive amount of replay memory is useful. Simulations have shown that an increase in at least one or both mentioned parameters does not significantly improve the results, and it prolongs simulation time. A higher temperature value indicates a high algorithm operating stability.

### 4.2. Results of the Experiment

**Figure 5:**
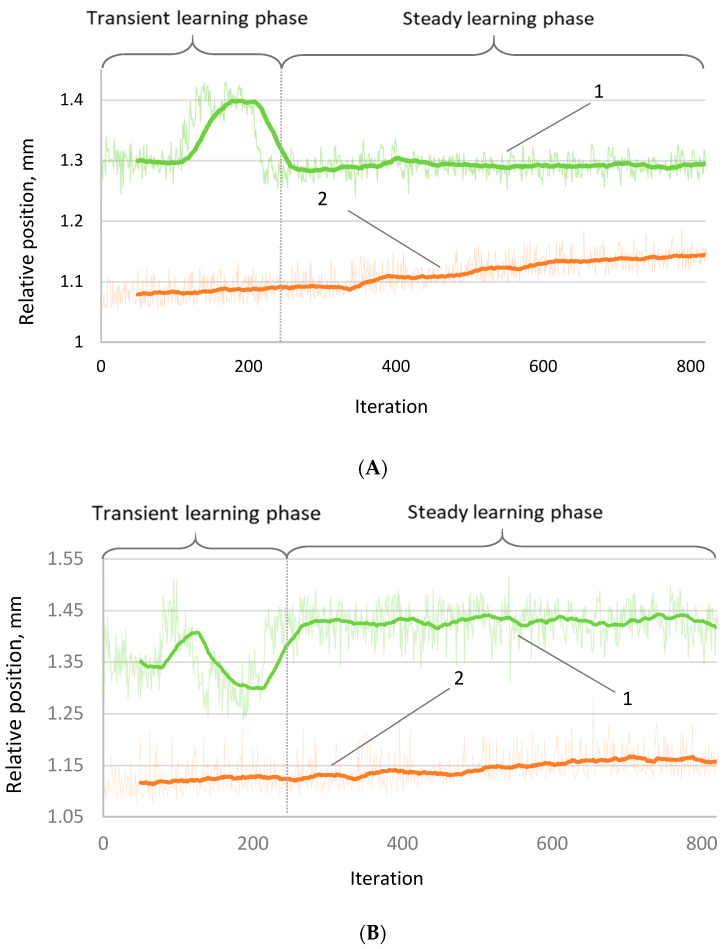
Variation of positioning of the final coordinates of (**A**) *y*-axis; (**B**) *z*-axis. 1—with correction and 2—without correction.

Robot positioning accuracy was evaluated using the algorithm parameters determined from the simulations. The dependency of relative position vs. the number of iterations shows how the position of the robot end effector changes during 800 motion cycles (Figure 5). It is seen that internal sensors do not notice the position drift when the robot works without the correction. The positioning deviation (curve 2, without correction) has practically the same amplitudes and trends in both y- and z-axes: 0.08 mm (from 1.10 to 1.18 mm) in the *z*-axis and 0.07 mm in the *y*-axis (from 1.08 mm to 1.15 mm). This difference might be explained by the fact that the load carried by the robot affects the movement of the robot mainly in the vertical direction.

The dependencies of the corrected position vs. iteration (Figure 5) show a completely different trend than the uncorrected case: large variations in position coordinates in the learning position of approximately 220 iterations. This behavior is highly expected because the algorithm at the beginning of the training performs “exploration”. At the end of this stage, there was a drastic change in the accuracy characteristics, and these coordinate values became much more stable. As in the case of uncorrected motion, the amplitude of fluctuations in the vertical axis is larger than in the horizontal axis. However, the adjusted positioning shows many more stable trends in the process perspective—both coordinates remain approximately constant.

**Figure 6:**
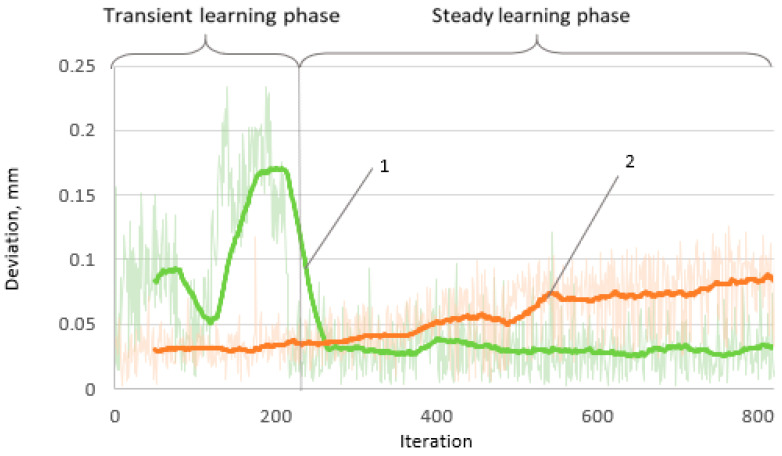
Variation of positioning deviation (error) with respect to the number of iterations: 1—with correction and 2—without correction.

The absolute error in terms of the number of iterations used to determine the robot’s positioning accuracy changes over time (Figure 6). As was expected, the positioning deviation increases with the number of iterations, and significant random error value fluctuations are observed in the short run.

The position deviation graph shows a distinct picture of the ML outcome from the original state. Here, after the end of the transient learning phase, when the steady learning phase begins, it is obvious that the value of the error remains approximately constant and is equal to that which occurs at the beginning of the uncorrected positioning. Such a situation indicates that the algorithm successfully eliminates positioning drift, although the mean error value remains similar to that which appears at the very beginning of the uncorrected positioning.

## 5. Discussion

There are a few algorithms that have been used for similar research, and each one has its own set of approaches that focuses on distinct factors. More detailed information about the methods and their achievements is provided in Table 1. Our research brings fresh results for machine learning integration into the robotic system as the strategy to increase robot positioning accuracy.

Our key finding is that applying different parameters to the ML algorithm helps us separate the relevance of certain characteristics and choose where we should concentrate our efforts. The importance of the algorithm setup parameters to achieve better learning procedure results is shown in Figure 4. This analysis might save time and resources in future studies, as well as improve the efficiency of the research.

Nevertheless, the proposed methodology faces limitations, too. Initially, this procedure is useful in case of a small number of points of interest in the trajectory. A considerable amount of data will require the entire mapping of the robot workspace rather than positioning error compensation ad hoc. The proposed ML procedure focused on the individual case of position, robot configuration in this position, and carrying load or end-of-arm tool.

The advantage of this methodology is that it is easy to use in the installed robot environment, where a particular task is processed, and the learning process brings an optimal value of robot position compensation for the analyzed case. Running the procedure does not require a ML professional; this is affordable for robot operators when the general script for the learning process is uploaded into a robot control system. Such a situation is advantageous in the real operation environment, especially for production process adjustment after its changes.

Although the principle of positioning error compensation is similar everywhere, the methods for achieving the result are very different (Table 1). Only similar robotic systems, signal fusion, and algorithms can be compared. The two closest studies to our method performed on 6 DOF robots with an image recognition system using deep reinforcement learning yielded an accuracy improvement of 20% and 50.3%, respectively [37,39]. Our algorithm reached a 66.6% improvement in positioning accuracy, decreasing the absolute positioning error value from 0.09 mm to 0.03 mm. 

As a scope of this research, the activity mainly focuses on one aspect of the robotic accuracy issues. A few further steps toward a multifunctional solution are required to make these results more applicable. Specifically, this research develops an algorithm for compensating position errors using visual control, performed by the optical sensor, which requires the installation of a camera with corresponding optics, with a magnifying ratio not less than 50×. During this ML process, the algorithm is constantly searching for the best path to reduce positioning errors after learning; therefore, the final number of loops does not exist. Continuous procedure during its run decreases error, but the best-achieved result with the time passing fluctuates due to process exploration. Therefore, the best learning result could be chosen separately from the learning history. On the other hand, the continuous learning mode loses a bit in positioning accuracy but remains flexible for the compensation values adjustment in case of external condition change (loads, thermal conditions, etc.).

Future studies could focus on eliminating the necessity to add visual control into the ML algorithm. This could be accomplished by locking/freezing compensation coordinates discovered without the use of visual sensors. To eliminate the constant need for a camera, it is possible to use distance sensors or their array and keep feeding the algorithm with live data from them together with internal robot sensor data. Future robotic systems possibly will be supplied with an internally installed ML technique using externally mounted sensors, indicating absolute positions of the robot’s trajectory points of interest.

## 6. Conclusions

This research provides an attempt to improve robot positioning accuracy using ML techniques. The implemented deep q-learning algorithm demonstrated efficiency by decreasing the robot position error from 0.09 mm to 0.03 mm. This result appeared as an outcome after approximately 800 iterations of the training process.

The newly created methodology improves the reliability and accuracy of robot positioning and decreases the duration of the ML procedure. This algorithm developed on the workbench allows accounting control of an unlimited number of variables per iteration with the proposed ML algorithm. This methodology is realized through the graphical user interface, which allows real-time monitoring of the procedure progress and control.

Nevertheless, the experimental learning process took a long time; therefore, there is an interest in and space for ML process simulation, which increase learning process efficiency. For this purpose, the original robot position deviations on the required point were stored and later used as simulation input. The developed methodology for simulation with many input parameters uses a special neural network. The simulation process lets us choose optimal activation and optimizer functions as well as the size of replay memory and the value of temperature. Moreover, after excessive trials, we defined the optimal size of the artificial neural network; it took one hidden layer with 37 neurons. The values obtained from the simulation were validated experimentally and confirmed initial assumptions of the proposed methodology. The robot positioning drift in the target point was efficiently compensated for externally without access to the internal robot control system.

The developed methodology of the online ML procedure is useful for all types of industrial robots. It is unrelated to the robot control system since it runs on independent external hardware. Our findings create a base of user-friendly and in-place online ML procedures that need adjusting to the level of the end user. Future research includes the analysis of different types of ML algorithms, the development of sensor fusion methods for online robot accuracy control, and further development of the algorithm focused on improving the accuracy of the entire robot trajectory.

## Figures and Tables

**Table 1:** Summary of research focused on trajectory generation and its accuracy.

Aims	Methods	Hardware	Achievements	Ref.
To improve the accuracy of the welding robot	Calibration based on deep reinforcement learning.	Yaskawa MA1-440 with controller DX200, laser vision sensor,	Achieved control error of less than 0.8 mm	[37]
To develop an open access dataset to verify robot calibration algorithms.	Levenberg–Marquardt (LM) algorithm and extended Kalman filter (EKF)	ABB IRB120 robot	The maximum positioning error is decreased by 68.07%	[38]
To reduce the absolute position error of robots	Machine vision and neural network	Hyundai Hi5 (HA006 model) 6-axis industrial robot, pneumatic gripper, laser measurement system, camera	Positional error reduced by 50.3%, reaching its absolute value equal to 0.029 mm	[39]
To improve celerity and accuracy of positioning for the spatial pose of the delta robot	Basic and optimized BP neural networks	MATLAB simulation	Delta robot system can achieve 97.75% accurate positioning with ± 0.05 mm tolerance	[40]
To develop a positioning error prediction model based on an extreme learning machine algorithm	An extreme learning machine algorithm	KUKA KR210 R2700, a laser tracker, and an accompanying spherically mounted reflector (SMR)	The accuracy of the robot was improved by 75.89% and 80.93%	[41]
To develop a system for automatic segmentation of the spine, pedicle identification, and screw path suggestion for use with an intraoperative 3D surgical navigation system.	Automated model-based approach. Accuracy was evaluated by comparing automatic segmentation to the manually outlined reference surface on Cone-beam images.	–	Success rate achieved of pedicle screw planning accuracy equal to 95.4%	[42]
Integrate accuracy enhancement method for a Cable-Driven Continuum Robot (CDCR)	The kinematic model and data-driven Gaussian Process Regression technique	Prototype of the CDCR, Qualisys Track Manager with six cameras and an industrial PC	Position and orientation errors reduced by 68.72% and 51.74%	[43]
To develop a method for complex robot inverse kinematics calibration	Inverse kinematic model based on multilayer perceptron	“Sina” surgical robot, infrared tracker	After calibration, positioning and orientation accuracy improved by 53% and 43%, respectively	[44]

**Table 2:** Summary of research on robot grasping technology enhancement using machine learning.

Aims	Methods	Hardware	Achievements	Ref.
To develop an image positioning and identification system for coal and gangue sorting robot	Least squares support vector machines	Industrial computer, V-GE502GC-T-CL Camera, MV-LD-12-10 M-J lens, robotic manipulator, belt conveyor	88.3% identification accuracy of the coal and 90.0% of the gangue sample	[47]
To build a robotic system that integrates grasping, vision, and motion planning to be able to pick items from a shelf to specific order boxes	Combination of machine learning and conventional feature-based strategy	Two lightweight UR5 robot manipulators, 3 stereo cameras, and 2 custom-built grippers	The system was able to pick 10 target items correctly in around 8 min	[50]
To incorporate force/torque information into reinforcement learning	Iterative Linear-Quadratic-Gaussian algorithm	Rethink Robotics Sawyer robot	Results show that using force/torque data, assembling accuracy of precise components could be increased	[46]
To develop a method combining a quality inspection system and process control	A convolutional neural network and computer vision	Kuka KR120 robotic arm Keyence LJ-7080 laser profilometers	A system able to detect defects and provide their quantitative characteristics	[45]
To develop a method for complex-shaped object position estimation after grasping	Machine learning-based classification method	A robotic arm equipped with a parallel gripper	The presented approach can be used as a good solution to overcome the possible uncertainties during the execution of a grasping task.	[22]
To develop a method estimating the geometric primitives of multiple circles in the 3D space for robot-assisted industrial automation	Multiple circular contours extraction, Maximum Likelihood Estimation SampleConsensus (MLESAC), Rodrigues formula, Delaunay triangulation, hierarchical clustering	KUKA KR 6 robot, AccuProfile 820-60 laser, linear motion system Rexroth Bosch	The method successfully implemented automation of the riveting of the fastener components on an aerospace structure	[51]
To implement semantic tasks, reach to grasp method for the industrial robot	Semantic grasp planning, model-based trajectory generation	Kinect depth sensor, 7 DOF Light Weight KUKA robot, WSG 50 parallel jaw gripper	Object discovery accuracy 95.8% Grasping accuracy 81.2%	[52]

**Table 3:** Summary of research on sensors and instrumentation in robotics.

Aims	Methods	Hardware	Achievements	Ref.
To compensate for undermined calibration values, sensor movement latency, and displacement offsets of IMU	Multilayer perceptrons, deep neural networks	IMU and IR sensors	69% reduction in tracking errors	[54]
To improve the accuracy of the force/torque sensor	Linear regression, Support Vector Regression	High dynamic range F/T sensor based on flexure mechanism	Accuracy has been improved using time-series data for sensor calibration	[56]
To calibrate augmented reality device using 3D depth sensor data	Neural network based on the VoteNet architecture	Microsoft Hololens, Kuka Mobile Youbot, Visual Studio 2019	Elimination of external tools used for augmented reality data calibration	[57]
To develop a methodology to detect and localize external contact	Random Forests and multilayer perceptrons	Proprioceptive sensors (joint positions, velocities, and one-dimensional (1D) joint torques. Kinova Jaco 2 manipulator	The time constant to detect contact equals 0.005 s in cases with a high contact force gradient	[58]
To improve the accuracy of IMUs used for position tracking	ML regression models based on long short-term memory	Xsens Avatar. 17 IMU’s	The proposed method ensures a lower average error of position tracking	[53]
To develop a fault prediction system for industrial robots	Gaussian mixture model-based unsupervised fault detection framework s	Industrial robot, current sensors	Prediction of gear wear faults in the robot with higher than 96% accuracy	[55]

## Data Availability

The data presented in this study are available on request from the corresponding author.
